# Ten simple rules for success as a trainee-led outreach organization in computational biology education

**DOI:** 10.1371/journal.pcbi.1013281

**Published:** 2025-07-11

**Authors:** Jennifer Blanc, Margaret C. Steiner, Lauren E. Blake, Elizabeth Gibbons, Mariadaria K. Ianni-Ravn, Roxroy C. Morgan, Suzanna Parkinson, Christian Porras, Ethan Zhong

**Affiliations:** 1 Department of Human Genetics, University of Chicago, Chicago, Illinois, United States of America; 2 Committee on Genetics, Genomics, and Systems Biology, University of Chicago, Chicago, Illinois, United States of America; 3 Department of Cell and Developmental Biology, Northwestern University, Chicago, Illinois, United States of America; 4 Committee on Computational and Applied Mathematics, University of Chicago, Chicago, Illinois, United States of America; 5 Center for Disease Neurogenomics, Icahn School of Medicine at Mount Sinai, New York, New York, United States of America; Carnegie Mellon University, UNITED STATES OF AMERICA

## Introduction

In the modern era of biology, essentially all scientific research involves computation in some form [1]. Accordingly, biology curricula from the undergraduate to graduate level are increasingly incorporating programming, math, and statistics into their curriculum [2–4]. However, this emphasis on computational skills is not reflected in most high school science classes, particularly in underserved communities [5–7]. This disparity has consequences, as post-secondary students with no prior exposure to programming are more likely to drop out of programming classes at the college level than peers with exposure during high school [5,8]. Prior programming exposure is also correlated with socio-economic status [7]. Therefore, it is reasonable to suggest that differences in exposure to computational science contribute to disparities of access and representation in the scientific community.

Addressing the exposure gap across states, school districts, and grade levels is complex. A wide variety of programs designed to teach programming skills exist at the K-12 level, but many barriers—most notably, resource and time limitations on both teachers and students—have prevented uniform adoption [9]. Additionally, these programs vary substantially in outcome, with some research suggesting that grassroots programs that emphasize broader skills beyond just basic programming and connect digital literacy to issues important to the intended recipients produce more positive outcomes [10]. If done thoughtfully and in collaboration with primary educators, universities are uniquely suited to help address the exposure gap on a local level: biology departments house a wide variety of scientists with computational skills, many of whom are interested in connecting with their local communities and sharing their knowledge.

With this in mind, we, a group of trainees at The University of Chicago, started University of Chicago COMputational Biology Outreach (UC COMBO) with the goal of contributing to K-12 computational biology education in Chicago. Our mission at UC COMBO is to “demystify” coding and quantitative science concepts that may initially seem daunting to aspiring life scientists in our community. We aim to inspire local students by showcasing numerous educational and career opportunities in computational biology and emphasizing the critical importance of computational skills across various disciplines within life sciences. As service to our mission, we have hosted or participated in 19 events, including 13 workshops that we’ve taught at local middle and high schools on the South and West sides of Chicago, and six community-centered events aimed at getting local students of all ages excited about STEM.

At UC COMBO, we have two goals for each workshop or event that we participate in, which are aligned with best practices identified by education researchers [11–14]. First, we aim to demonstrate why computational skills and thinking are important to solving biological problems. We understand that we will not produce expert programmers in a two-hour workshop, but believe there is value in introducing these topics and sparking students’ interest. To meet this goal, we ensure that all of our workshops, which are publicly available, center on the application of concepts rather than syntax or memorization. Additionally, we provide a list of online programming education tools for students who are interested in learning more. Second, we aim to provide students an opportunity to meet working scientists and learn about career paths in computational biology. Every workshop includes not only a hands-on computational biology activity, but also a talk and/or group discussion about higher education and STEM careers led by UC COMBO instructors. During this session, we discuss some of the research questions we are working on, what we do on a day-to-day basis, and how we became scientists. For many of the students we have connected with thus far, it was their first time meeting a scientist in person, and we have found these conversations to be just as important as the lesson itself. Keeping in mind our capacity as a trainee-run organization, these goals are both achievable and have components that are testable via feedback from both students and teachers.

**Fig 1:**
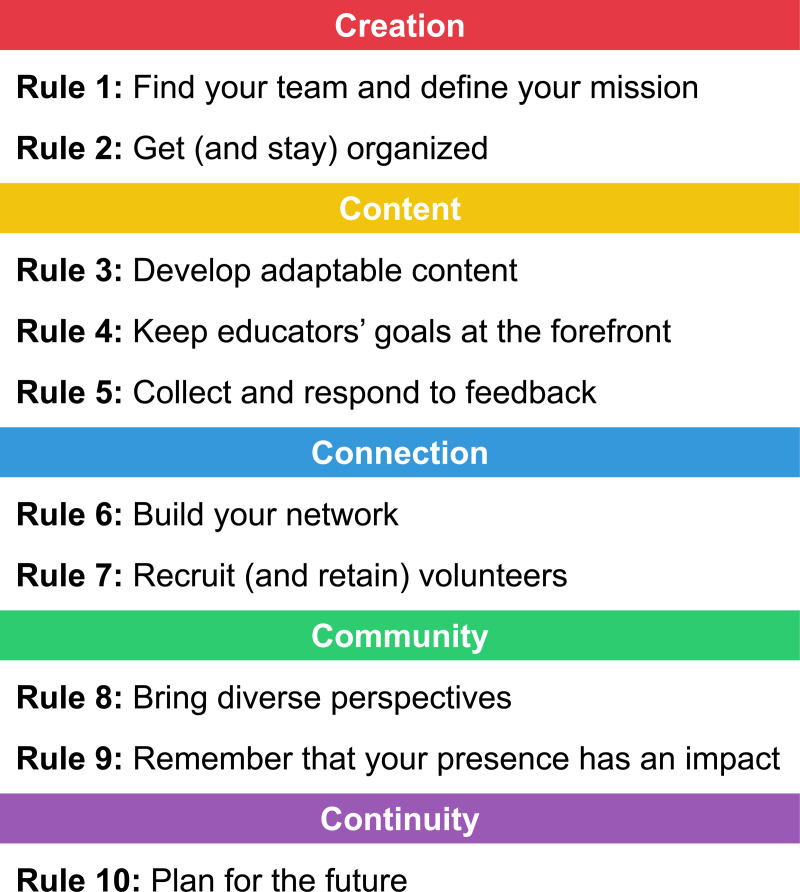
Overview of the ten simple rules. Each rule aligns with one of five themes: creation, content, connection, community, and continuity.

Starting a computational biology outreach organization has been a challenging yet extremely rewarding experience. Below, we provide 10 rules for success that have helped us build and expand UC COMBO (see also [15] for suggestions on effective process design). It is important to acknowledge that we are not professional K-12 educators or outreach professionals, but rather are a group of students and postdocs who are passionate about computational biology and connecting with our community while balancing our research commitments. Yet, we have learned a great deal about outreach at the K-12 level. In the spirit of open access, collaborative science, and promoting science education, we hope that sharing our experiences and the lessons we have learned will help other trainees to get started with computational biology outreach. Below, we condense our experiences and suggestions into 10 Simple Rules which we believe are broadly relevant to outreach across all STEM fields. These rules are organized into five themes: creation, content, connection, community, and continuity (Fig 1).

### Rule 1: Find your team and define your mission

Creating a team is a critical first step in starting any outreach group. In our case, we looked for motivated students and postdocs within our university ecosystem who were interested in education and community service and willing to devote time to launching the organization. Time commitments for each team member will vary greatly depending on the size and goals of the group, however, at the start it is ideal for group leadership to have 1–2 h available per week to meet, and 4–6 h available in the week leading up to your first event. It is important that this group communicates well and aligns on the organization’s mission.

To start defining your mission, we recommend asking yourself the following three questions:

1. What type of outreach are you interested in doing? Would you like to teach at the elementary, middle, or high school levels? Do you want to plan one big, yearly event or several smaller events every semester? Do you want to focus on any particular subject matter?
2. What do educators in your community need? What type of programming do teachers and other educators (e.g., after school program leaders) think is needed? What other outreach groups exist at your university or in your community, and what programming do they provide? Are there “gaps” in current outreach offerings that your group could fill? We encourage you to connect with local educators at this stage in order to learn from the source about what would most benefit your community (see also Rule 4).
3. What will students get out of your program? Would you like to teach them a new skill? Get them excited about science? Learn about future career options? All of the above?

As an example, when starting UC COMBO, we were interested in holding short workshops for the middle- and high-school grade levels, as well as organizing booths for local STEM fairs. After meeting with local teachers and after-school programs, we decided to focus more of our energy on middle school programming, as there were already several groups offering outreach to high schools but few for middle schools. We decided that we wanted students to learn why computational skills are important (in biology, and elsewhere), rather than focusing on teaching syntax, and wanted to introduce different educational opportunities and career paths in STEM.

We encourage you to think about how your answers to the questions above fit together. Each is important to the success of your program. If you are not interested in the work you are doing, you will not be motivated. If the community does not have a need for your program, you will struggle to find opportunities to get involved. And, of course, the goal at the end of the day is always for students to get something out of your outreach. We recommend writing a mission statement early on, but do not be afraid to change it as your organization evolves.

### Rule 2: Get (and stay) organized

**Fig 2:**
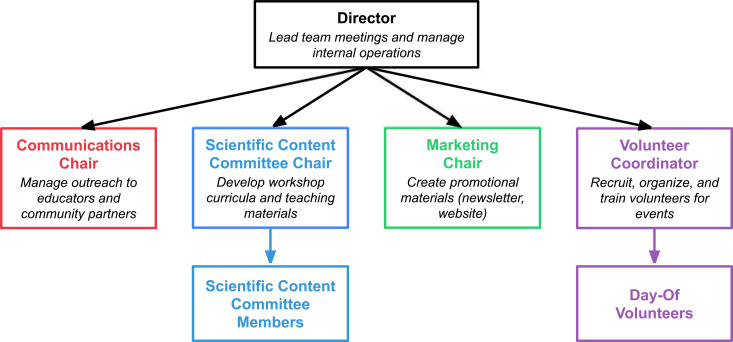
Example of an organizational structure used by UC COMBO.

Once you have found a committed team and agreed on the mission of your organization, it is important to start getting organized. Here, we will discuss what has worked well for our group. First, we found that designing an organizational structure such that each member of the group has a well-defined (and non-overlapping) role was key to our productivity (Fig 2). Especially at the start of an outreach initiative, the group is likely small and time is limited, so it is helpful to “divide and conquer” on important tasks. This structure will likely change over time. In our experience, we found that having one person each in charge of school outreach and logistics, workshop creation, and internal operation/organization development was a natural fit at the start, and as our group expanded, we added roles relating to volunteer coordination and marketing/graphic design. We also expanded scientific content creation into its own committee. Exactly how this setup looks will differ depending on your specific goals and team, but having clear responsibilities will help to ensure that all of the important tasks are being taken care of.

If your organization will require funding for any supplies or stipends for members, we recommend reaching out to faculty members, academic departments, or your university’s community service and/or student organization offices. Funding needs will vary greatly depending on the goals of your organization. As an example, COMBO was supported for several years by discretionary funds of a professor in the Human Genetics department, but we recently obtained status as a Recognized Student Organization which opens up additional funding and support avenues.

Once you have set up your organizational structure and acquired any necessary funding, it is important to establish good habits for communication and documentation. For instance, setting up a recurring meeting time to touch base on activities and plan events will help keep goals on track. Setting up a shared digital folder to store meeting notes, event information, and workshop materials will help to keep track of important information over time. We have found it helpful as well to maintain an “onboarding” document detailing our organizational systems and procedures to help with transitions to new teams over time (a template is provided as supplemental material; S1 Appendix). Lastly, we encourage your group to take time to set group norms and expectations around work style and practices in order to promote accountability as well as to foster an accessible and inclusive environment.

### Rule 3: Develop adaptable content

After getting organized, we recommend turning your attention to designing content for your outreach activities. Your mission will help you create a framework for the key points you want students to take away from every lesson you create. But, there are still several important decisions to make. Since you will likely be wanting to reuse lessons for multiple different events and workshops in the future, it is important to design activities such that they are adaptable to different amounts of time available, numbers and ages of the students, and availability of materials.

**Fig 3:**
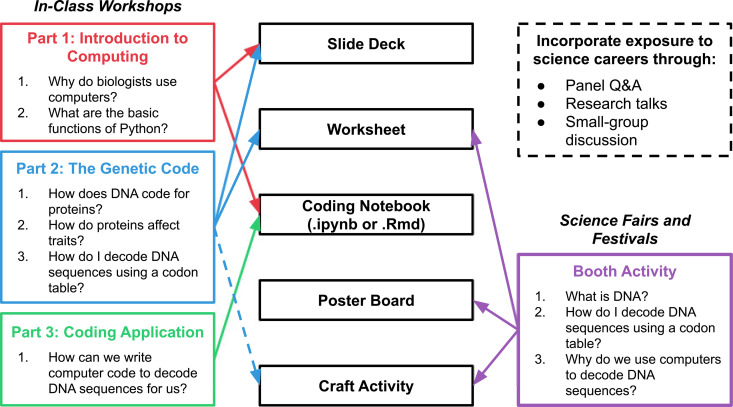
Schematic depicting two forms of our genetic code workshop (in-class workshops and science fairs/festivals) and materials used for each.

The first question to ask is: what do you want to teach? We suggest choosing a problem in biology or medicine that is well-known, engaging, and accessible for multiple age levels. It is especially helpful to choose topics that are aligned to the local public school curriculum (e.g., the NGSS guidelines used in 20/50 states). This will make it easier for teachers to incorporate your outreach activities into their classes. Plus, students will be more engaged when the topic is familiar. For example, one of our favorite workshops centers around the genetic code (Fig 3). We hold this workshop at a local middle school at the same time every year to coincide with that school’s sixth-grade science syllabus. We start by recapping information we know the students have seen, e.g., how to read a codon table manually and translate a nucleotide sequence into amino acids. Next, we show the students how to do this using Python and explain how this computational approach enables researchers to analyze vast amounts of genetic information (e.g., “How long would it take you to decode the entire human genome by hand?”). While students have already been exposed to the central dogma, the concept of using computers to translate the sequences is new to them. We find that layering on the computational material with familiar biological concepts makes it more accessible.

Having chosen a topic, think about how you will format your lesson. It has been beneficial for us at UC COMBO to develop modular content that is easily adaptable to different situations. Every teaching opportunity has different considerations: class times are variable, students have different backgrounds or education levels, and certain resources are not always available. Our workshops have typically fallen into three different categories: (1) science-fair booth activities, (2) middle school classroom workshops, and (3) high school classroom workshops. These last two categories include both workshops given to an entire class or grade-level during the school day and after-school workshops for specialized clubs (i.e., science club). For the first category, we frequently employ hands-on activities (e.g., crafts and worksheets) developed to get students engaged and excited about the topic, as well as introduce key concepts. For the latter two categories, we also prepare short lecture presentations on both biology and coding topics as well as interactive coding notebooks to use for the programming portions of our workshops (we format these as Jupyter notebooks and host them on Google CoLab). This setup makes it easy for students to open their laptops and start coding immediately without having to install any software. For each of the three workshop types above, we can then combine these activities in different ways depending on the amount of time available and age of workshop participants.

### Rule 4: Keep educators’ goals at the forefront

Your community partners (i.e., schools, museums, libraries, and community centers) will often be constrained in the amount of time, money, and resources they have to meet specific academic goals. It is therefore important to keep the educator’s needs and goals in the forefront and ensure that your program is aligned with them.

Practically, what does it look like to center your efforts on educators’ goals? As you begin to plan your outreach activities, look up any education standards or guidelines that apply to the teachers in the school you will be visiting. When you approach educators, highlight how your outreach will help students meet those standards and support their classroom learning. Often, the topics in a workshop can be interchanged to better align with interests of the class and/or curricular goals. For example, our adaptable content (see Rule 3) includes a workshop on the basics of GWAS. This workshop supports students in meeting the NGSS standard HS-LS3 regarding the Inheritance and Variation of Traits. Depending on whether the class has recently learned about agriculture or human disease, we can adjust the target trait of the simulated GWAS in our coding notebook (e.g., crop yield vs. LDL cholesterol).

Even the best-laid plans will need to be adjusted according to educators’ needs, so be open to new setups. It usually takes only a few questions to determine how you should structure your outreach event. For example, you might ask:

- From a provided overview of the content you have prepared, which topics do they prefer that you teach?
- What will they be teaching around the time you visit, and how can you modify what you teach to support their learning objectives?
- What class size should you prepare for? Do they prefer that you work with one large group of students or several smaller groups?
- How much time do you have with the students? Will that time be broken up into shorter sessions or over several days?
- If you plan on using specific equipment (laptops, a projector, etc.), do they have those materials or should you provide them?
- Are any web-based materials you plan to use available on their WiFi? Educational centers sometimes block innocuous websites—check this beforehand!
- Are there any special circumstances that you need to be aware of? For example, there may be students who are learning English as a second language or who have a learning disability. For our genetic code lesson, we have both an English and Spanish version of the background slides and coding notebooks prepared, as one of our recurring events takes place in classrooms that include some primarily Spanish-speaking students. The teachers alerted us beforehand, and we were able to translate the material with the help of volunteers in our network.

When the time comes to actually go and do your outreach activities, make it easier on educators by handling as many of the logistics as possible. Their jobs are already difficult enough, so make every effort to avoid complicating them!

### Rule 5: Collect and respond to feedback

Once you start holding outreach events, feedback is a crucial tool for growth and improvement. Gathering input from students, educators, and volunteers will help assess the impact of your programs and inform continuous improvement efforts. It also demonstrates to your community that you value their opinions and are committed to refining your program. Finally, for groups seeking funding, such metrics can be useful for demonstrating impact and establishing credibility. In this section, we provide some guidelines on feedback collection based on our experience.

1. Collecting Feedback: There are multiple ways to collect feedback after an outreach event, and we have found it helpful to implement several of these methods in tandem. First, we provide teachers with post-event surveys to ask if they felt the material was relevant, engaging, and aligned with their students’ learning needs, as well as ask for suggestions for modifications that can be implemented in future workshops. To maximize participation, we design our feedback forms to be concise—typically comprising 5–7 questions that can be completed in under five minutes. In addition to feedback from teachers, collecting feedback directly from students is invaluable. Short, anonymous feedback forms, featuring a mix of yes/no questions, Likert scales (e.g., rating a lesson from 1 to 5), and open-ended questions, are all useful. Asking students what they learned and what their favorite and least favorite part of the lesson was yields important data that can help you fine-tune your material and delivery.
2. Creating a Feedback Loop: Collecting feedback is only the first step; the most important part of the process is using that information to improve future workshops. After every outreach event, take the time to review all the feedback as a team. Documenting the feedback in a centralized place (such as a shared document or spreadsheet) will help your team keep track of recurrent themes, allowing you to quickly identify what aspects of your workshop are working well and which ones may need adjustment.
  Beyond documentation, it is critical to have a system in place for acting on feedback. The feedback process should be cyclical: gather information, discuss as a team, implement changes, and then gather feedback again in an ongoing loop of refinement. One specific way that feedback has shaped UC COMBO’s programming is in adjusting the technical level of our workshops. After our first few events, we noticed that some students were getting lost during the coding portion of our workshops, particularly if they had little to no prior experience with computational tools. In response, we simplified the main content and then built in challenge problems for more advanced students. This adaptability helped make the workshops more inclusive and allowed students of different skill levels to feel comfortable engaging with the material.
3. Closing the Feedback Loop: Responding to feedback isn’t just about internal adjustments—it’s also about maintaining open communication with the educators and institutions you work with. After implementing changes based on feedback, let your collaborators know what actions you’ve taken. This could be as simple as a follow-up email to teachers, sharing how their input has shaped future iterations of the workshop. This not only shows your partners that their opinions are valued but also builds stronger relationships and trust, making them more likely to work with you again in the future.

Finally, closing the feedback loop also means sharing success stories with your team. Recognizing what went well helps maintain morale within the outreach group and can guide future planning. By highlighting successful elements, your group can build on its strengths while continuing to address areas of improvement.

We also encourage you to understand the feedback that you get within the context of your event. For example, we have found that, perhaps unsurprisingly, not all of the students we interact with enjoy learning how to code. And, that is ok! Our goal is not to convince *every* student to major in computer science or become a computational biologist. Different students will have different interests, and it’s alright if computational biology isn’t necessarily up their alley. Our goal is to make sure that these students still learn something from our outreach and have fun in the process.

Beyond immediate feedback, we suggest employing a combination of pre- and post-workshop assessments to gauge knowledge acquisition, track participation rates, and monitor long-term engagement through follow-up surveys.

### Rule 6: Build your network

One of the hardest tasks when getting UC COMBO off the ground was connecting with local educators and scheduling our first set of events. As graduate students and postdocs who often have only recently moved to our city, our roots in the community outside of the university can be limited. With the caveat that not all situations are the same, in this rule, we share some of the strategies that have worked for us in terms of building our network in the community.

The first lesson we learned is that cold emails are not the best strategy to reach educators. Teachers are busy and often don’t have time to answer cold emails, especially ones from a new, unfamiliar organization. Instead, we found that leveraging personal connections was the key to scheduling our first few workshops. We asked all of our members, along with people in our department’s ecosystem, if they knew any local teachers (even if they are not science teachers) that they could connect us with. In fact, we set up our longest partnership with a local middle school because one of our founding members had a friend who used to be a teacher at the school and was kind enough to introduce us to the current team of sixth-grade science teachers. These teachers were eager to work with us to host a workshop that aligned with their curriculum, and we have since repeated this workshop every year. Overall, we found that emails which included a personal connection were much more likely to get a response, and educators were much more likely to take a chance on us—or introduce us to other teachers who would.

We also learned that it is imperative to tap into the local outreach ecosystem. For us, this involved signing up to give demos at two different local science fair events. Local STEM fairs and events are also fantastic for networking with other like-minded individuals and groups. Alongside our activity, we brought fliers about our organization and talked to all adult attendees about what we do. Many attendees are teachers or parents looking for enrichment opportunities for their local schools and were excited to connect with us. We have developed several ongoing relationships this way which have led to recurring events in the community. Both personal connections and connections to the existing outreach ecosystem helped us identify the gaps in current outreach programming and understand the long-term needs of our community.

Finally, we encourage you to leverage new relationships into additional connections and set up recurring events. We often find it helpful to ask educators in our network to connect us with colleagues who may be interested in our workshops—a personal recommendation can go a long way. Additionally, once we complete an event and ask for feedback, we always ask our point of contact if they would be interested in us returning next year when they have a new group of students. This has been very successful in establishing continuity and has allowed us to improve our workshops while building lasting partnerships.

### Rule 7: Recruit (and retain) volunteers

We know that recruiting volunteers for a newly established outreach organization can be challenging at times, and maintaining engagement is equally difficult due to natural turnover. In this rule, we share a few strategies that have helped us build and sustain a group of about 30 volunteers, even as individual participation has varied over time.

1. Get the word out: Utilize departmental platforms, such as mailing lists and bulletin boards, to promote upcoming events and attract potential volunteers. Participate in your university’s student organization and volunteer fairs (this also helps to meet others in the outreach ecosystem). At the beginning especially, it helps to cast a broad net. When promoting an event, make sure to provide clear information on the expected time commitment, types of tasks involved, and the level of experience required so that individuals can make an informed decision about whether volunteering aligns with their availability and interests.
2. Use your academic network: If there are particular people in your department or program with subject matter expertise in the material you are teaching, it can be very effective to reach out individually.
3. Communicate: Effective volunteer recruitment also involves clear communication with volunteers before and after events. We recommend scheduling in-person meetings with the volunteers prior to an event in order to provide a detailed walk-through of logistics and responsibilities. At this meeting, we find it useful to cover some of the basics of inclusive pedagogy practices and role-play some common scenarios. In addition, these meetings are often an excellent time to address strategies volunteers may use to deal with more sensitive questions on the lesson topic. For example, in our basics of GWAS workshop, we are always careful to instruct our volunteers against making generalizations which imply genetic determinism of the complex traits in our discussion. After the event, be sure to generously acknowledge the contribution of volunteers and keep them informed about future events and opportunities. Keep in mind that if volunteers enjoy their experience, they will be more likely to tell their friends and coworkers to volunteer too!
4. Recognize and retain: Retaining volunteers requires ongoing appreciation and engagement. Sending timely thank-you emails to volunteers, providing breakfast/lunch for volunteers, and reaching out with regular updates on your organizations’ accomplishments are all great ways to recognize the effort of your volunteers, foster their sense of belonging, and encourage them to participate again. If resources allow, we also suggest considering ways to recognize volunteers’ experience with teaching certificates or other formal credit through your university or graduate program. These strategies help volunteers feel valued and invested in the organization’s mission over time.

### Rule 8: Bring diverse perspectives

Representation matters, especially in STEM fields [16,17]. To support this, we recommend structuring volunteer opportunities in ways that are sustainable and equitable—for example, by providing leadership development and teaching opportunities in recognition of volunteering work. An environment which is inviting and rewarding to mentors from all identities, over time should foster a volunteer base of diverse ethnicities, genders, and other backgrounds, without placing the workload disproportionately on historically marginalized groups. Having a volunteer base with diverse backgrounds and experiences can also help you to better understand the needs of your students, which will in turn create a more inclusive environment for them. When students see role models who reflect their own identities, it can help them to feel more connected to their teaching group and subject matter [18].

Bringing in volunteers from varied career paths can also help students grasp the broad opportunities in computational biology. We have found that students are very interested in learning about what a PhD is, asking questions about colleges and majors, and what we do in our jobs on a day-to-day basis. For example, students are typically unaware that many PhDs are fully funded, and that our job involves more research than coursework. (In fact, one of the most common questions we get is, “How much homework do we have?”) Additionally, we spend time discussing scholarships, fellowships, and other programs that give students an opportunity to work in science labs. We try to expose students to the diverse career paths available to those who pursue advanced degrees in STEM, from academia to industry and beyond. Lastly, we have found that workshops benefit from including volunteers at different stages of their academic careers who can provide unique viewpoints on their experience.

### Rule 9: Remember that your presence has an impact

Once you have set up your group, developed your material, and now found volunteers to help you carry out your plan—we encourage you to think about what you represent when you go to a school or an event. As stated above, many of your students will not know any scientists outside of their science teachers, and even fewer will have interacted with practicing researchers. Outreach programs are not just about introducing scientific material but also about interacting with scientists. And, it has been shown that role models—especially those who counteract stereotypes about scientists—have a meaningful impact on success in STEM [16,18].

We have found that scheduling times for connection as well as more spontaneous communication can lead to meaningful interactions between students and researchers. For example, at the beginning of a workshop, we have volunteers spread themselves out in the room so that they can speak to the students in small groups and begin to break the ice. Having volunteers introduce themselves and share a few fun facts with the students is a great strategy. These interactions help convey that scientists are people too and can make students more comfortable, especially when asking questions later in the lesson.

We have also incorporated short “career talks” during many of our outreach events. During these talks, trainees or faculty discuss their academic journeys and the research questions they work on. Often, this will be students’ first exposure to university-level research and niche sub-fields of biology. We have seen firsthand that these talks spark invaluable discussions for mentors and mentees alike.

### Rule 10: Plan for the future

Having described our experience in creating how to create a successful outreach organization, we want to end by dwelling on what it takes for this success to *continue*. A unique challenge for trainee-run organizations is the inherent turnover in leadership as students graduate and postdoctoral fellows take new positions. Many of the rules outlined above will be helpful in planning for turnover (in particular, Rules 1, 2, and 7). In addition, we encourage leaders of trainee-run organizations to continuously recruit new members with an interest in outreach, train more junior members to take over their roles, and to document group procedures and contacts as thoroughly as possible. Obtaining support (financial or otherwise) at the level of a faculty member, department, or academic office, can also help to sustain the program over time. At the end of the day, the key to making any outreach organization last is having a mission and programming that everyone—including the trainees, educators, and students in the community—is excited and enthusiastic about continuing on.

## Conclusion

We hope that these rules have provided some insight into how to improve a current computational biology outreach program or given you the tools to start one of your own. We note that even though efforts made by individual groups and departments may seem small, these initiatives together can have a huge impact. Encouraging such “grass roots” efforts across institutions will help to train a generation of involved, community-minded scientists and expose more and more students to opportunities in computational biology and beyond.

Keeping this in mind, we want to end with one of the most important tips to keep in mind as you venture into science outreach: have fun with it! Outreach provides a valuable experience for both scientists and the community. For scientists, we invite others into our world and teach them about the curiosities that drive us, sparking interest and excitement in the audience. Also, as researchers, we are accustomed to waiting months (if not years) for a project to develop, but outreach provides instantaneous joy in seeing students learn and explore. Students, on the other hand, can experience science in a way that allows them to connect concepts and ideas with real-world applications and potentially be inspired to pursue science or a similar career path. As such, we have to remember that outreach should be a fun experience for everyone involved. When we engage with audiences, the joy and passion for what you do must be a given. Our excitement helps the students get excited about science, creating a powerful positive feedback loop that can leave a lasting impression long after your outreach has concluded.

## Supporting information

S1 Appendix Onboarding document template. (PDF)
