# Supplementary material for: Ten simple rules for success as a trainee-led outreach organization in computational biology education
Source: PLoS Comput Biol. 2025 Jul 11;21(7):e1013281. doi: 10.1371/journal.pcbi.1013281 (PMC12250629; doi:10.1371/journal.pcbi.1013281)
Supplement: S1 Appendix — (PDF) [file pcbi.1013281.s001.pdf]

Onboarding Document Template

Last updated: MONTH YEAR

Note: This document is a modified version of UC COMBO's onboarding document, with some information removed.

Mission Statement

To be customized to your group's mission. We provide the mission statement for COMBO as an example. Our mission is to demystify coding and quantitative science concepts that may initially seem daunting to aspiring biologists and life scientists. We aim to inspire students from diverse backgrounds by showcasing numerous career opportunities in computational biology and emphasizing the critical importance of computational skills across various disciplines within life sciences.

Internal communications

Slack

Leadership team communication occurs primarily through Slack. Provide a link to join Slack workspace and note names of channels (or similar for other platforms).

Account login information

Google/Gmail
Email
Password

Website
URL
Login Instructions

Github
Name of Repository
Login Instructions

Google Drive

All COMBO materials are organized in the below Google Drive folder: LINK

Mailing lists

- University-wide mailing list information

Leadership team contacts

Name	Email	Cell
First Last	Email Address	XXX-XXX-XXXX

Organizational Structure

To be customized to your group's needs. We provide our current structure as an example.

Overview

- Director: Name
  - Oversee COMBO's internal operations & leadership team to ensure goals and objectives are met
  - Identify opportunities for growth through internal (UChicago) or external partnerships
  - Schedule and run monthly Leadership Team meetings
- Communication Chair: Name
  - Coordinate with schools and teachers to organize events
- Scientific Content Committee Chair: Name
  - Lead the rest of the scientific content committee to create and organize content for events
  - Coordinates with the rest of the COMBO team about workshop content
- Marketing Chair: Name
  - Send out a newsletter 1x/quarter
  - Maintain the website
  - Create flyers (e.g. recruiting volunteers in elevators, for workshops, etc.)
  - Create marketing materials
- Volunteer Coordinator: Name
  - Recruit and organize logistics for event volunteers

Role Descriptions

Director

1. **Oversight of leadership committee:** The main responsibility of the Director is to oversee the leadership committee. This includes scheduling and leading regular committee meetings (including creating and sharing an agenda prior to each meeting). During these meetings, the Director will ask for updates from other committee members on ongoing activities and delegate any action items to members of the team. The Director should also keep in regular contact with other leadership committee members to ensure projects and event preparations are on track with all deadlines.
2. **Internal communications:** The Director will keep notes during/after meetings and share these notes with the leadership committee. The Director should also follow up on action items via Slack and send reminders prior to events and meetings.
3. **Organization development:** The Director is responsible for ensuring that COMBO continues to grow and improve in the medium- and long-term. As such, they should identify and carry out initiatives that serve COMBO's strategic mission (as an example, obtaining RSO status). The Director should seek out partnerships with relevant organizations on- and off-campus to assist with COMBO's activities (e.g., University offices, other student organizations) and will serve as point of contact for any partnerships.
4. **New member recruitment:** The Director is responsible for organizing new member/volunteer recruitment initiatives, typically during the Autumn quarter (e.g., presentations at orientation events, tabling the Broader Impacts Fair).
5. **Events/teaching:** It is expected that the Director is present for as many COMBO events and workshops as possible. The Director will also take a significant role in teaching at said events.

Communication Chair

1. **Communication with existing POC:** The communication chair is responsible for maintaining relationships with existing points of contact (listed below). This includes reaching out ~2 months before a proposed workshop time and working with them to schedule workshops/events.
2. **Outreach to new POC:** Besides scheduling recurring events, the communication chair is partially (alongside the diretor) responsible for making new contacts helping to grow UC COMBO within the existing capacity.
3. **Day of logistics:** As part of the planning of both new and existing events, it is the communication chair's responsibility to work with the POCs to plan the day of logistics. This includes gathering information about time to arrive, parking, checking in, number of students, academic background of students, making sure the POCs approve the lesson, set up of the room, technology available to students, and, most importantly, will the notebook work on the student's laptops.

4. **Teaching + Support:** As a committee chair it expected that the communication chair pull significant weight in regards to teaching the lessons and attending the events.
5. **Monitoring UC COMBO email:** The communication chair should regularly check the UC COMBO email and either reply to inquiries or pass the information onto the relevant leadership member.

Scientific Content Committee Chair

1. **Coordinate creation of new content:** The content chair is responsible for coordinating the efforts of all members of the Scientific Content Committee (SCC). This means leading the team to reach attainable goals, maintaining to-do lists, setting deadlines when needed, and delegating tasks among team members based on interest and availability. The SCC currently operates very democratically - decisions on where to take content and topics to explore is decided as a group. It is the chair's role to organize the group's efforts to be maximally effective.
2. **Manage existing content:** The content chair is responsible for keeping all existing and in-progress workshops organized on the UCCOMBO Google Drive and github. While this is also a team effort among all members of the SCC, it is the chair's role to maintain an organized system so content can be easily found and understood. It is extremely valuable to have all materials on hand and in working order so we can offer workshops when there is a demand with minimal changes to existing material.
3. **Review and provide feedback for content:** While the rules of content creation are very flexible, COMBO aims to keep content within the margins of K-12 learning standards. The content chair is responsible for keeping the team on target to provide fun and educational workshops to the desired demographic. This means reviewing and providing feedback for existing and in-progress content to ensure it is understandable and appropriate for a K-12 audience.
4. **Communicate between leadership team and content team:** The content chair will also keep the leadership team up to date on new content creation. In addition, the content chair also makes sure SCC members are informed about important happenings on the leadership team, upcoming events, and organizational changes.

Volunteer Coordinator

1. **Recruitment**: The volunteer coordinator is responsible for recruiting volunteers who have the skills and enthusiasm for introducing computational biology concepts to younger students. This involves reaching out to potential volunteers within COMBO and beyond, such as through campus-wide announcements.
2. **Training**: Once volunteers are recruited, the coordinator organizes orientation sessions to familiarize them with the workshop logistics, lesson plan, and tools or resources they will use during the workshop. This ensures that volunteers are well-prepared to deliver high-quality educational experiences to the audience.

3. **Support**: During the day of the workshop, the volunteer coordinator shall provide on-site support to ensure everything runs smoothly. This includes acting as a point of contact for volunteers if they have questions or issues before coming to the workshop and/or during the workshop session.
4. **Logging**: After the workshop, the volunteer coordinator should keep a record of volunteer participation for tracking progress and demonstrating the impact of the workshops over time.

Marketing Chair

1. **Branding:** The marketing chair ensures UC COMBO presents a cohesive brand: this includes designing a recognizable logo, developing color schemes, and creating visually appealing layouts for all materials.
2. **Promotional materials:** the marketing chair designs and orders banners, T-shirts, flyers, and other marketing tools for events. These materials will be used to attract volunteers, promote workshops, and highlight UC COMBO's mission at outreach events.
3. **Website**: the website should be regularly updated (for example, with leadership contact details, new workshop information or blog posts). It should function as a resource for volunteers, parents and potential collaborators.
4. **Newsletter**: the marketing chair is responsible for writing (quarterly) newsletter. These can act as a tool to highlight past events, share upcoming opportunities, and provide insight into COMBO’s achievements.

Committees

Scientific Content Committee

1. **Attendance at Content Committee meetings:** Members of the SCC are expected to attend biweekly meetings led by the SCC Chair.
2. **Creation of new content:** SCC members are expected to contribute to the development and testing of teaching materials including coding notebooks, worksheets, and lecture slides.
3. **Meet *hard* deadlines when needed:** Nearly all our deadlines for the content committee are soft deadlines. We try to do most of our content creation well in advance of planned or anticipated events, but occasionally, we have to make hard deadlines. It is important that content committee members are respectful of these and communicate if they need assistance in completing tasks before them.
4. **Maintain an organized and positive workspace:** Keep content in appropriate directories, give files informative names, don’t overwrite other’s work, be mindful of deleting things, and be respectful when commenting on other’s work and collaborating with other SCC members.
5. **Teaching responsibilities:** Interested SCC members are encouraged to participate in teaching during COMBO workshops.

Policies & Procedures

Meetings

Leadership team members are expected to attend once-monthly meetings. Scientific content committee members & chair are also expected to attend committee meetings, typically held biweekly.

Events

Leadership team & content committee members are expected to regularly attend COMBO workshops and other events (when possible).

Recurring Events

Points of Contact

School/Organization	POC Name	POC Title	POC Email	Typical timing
School	First Last	Grade, Subject (for educators)	Email Address	Month/Season

Event Descriptions

We encourage you to keep descriptions of all past events on hand. We provide an example below.

School A Workshops

We hold annual events at School A, typically in early December. We teach the Genetic Code lesson to the 6th grade classes. Our workshop is meant to complement the School A 6th grade science curriculum that is taught in late Nov./early Dec. The exact format depends on the preferences/logistics of the school & 6th grade science teachers. We have had better experiences when we are able to teach several smaller groups of students as opposed to all sixth graders at once.

New Member Recruiting

COMBO typically participates in the following new member recruiting activities in the Autumn Quarter:

- Event Name (POC: First Last, Email Address)

Log of Past Events & Workshops

Date	Location/ Event	Level	Lesson/ Content	# Students	# Volunteers	Notes
MM/DD /YY	Event Name	Elementary/Middle/ High School	Lesson	XX	XX	XX

COMBO Alumni

- First Last - Role at University, Position in Organization
